# Acceptance and Usability of a Web Application for Patient Care Level Classification in German Clinical Nursing Care: A Pilot Study

**DOI:** 10.1055/a-2753-9439

**Published:** 2025-12-11

**Authors:** David Powering, Nico Humig, Eva Rothgang

**Affiliations:** 1 Department of Industrial Engineering and Health, Institute of Medical Engineering, Technical University Amberg-Weiden, Weiden, Germany

**Keywords:** evaluation, mixed methods, Technology Acceptance Model, System Usability Scale, nursing documentation, PPR 2.0, web application

## Abstract

**Background:**

The German Federal Ministry of Health introduced the Pflegepersonalregelung 2.0 (PPR 2.0) to address the nursing staffing crisis. It establishes a framework to determine personnel requirements, ensuring adequate staffing. However, the required daily classification of patient care levels imposes a significant administrative burden on nursing staff. Digitizing this process may reduce documentation time and enhance efficiency, but effectiveness depends on usability and acceptance.

**Objectives:**

This study evaluates the acceptance and usability of a direct digitization of the analog PPR 2.0 classification catalog into a digital user interface—the PPR 2.0 Calculator.

**Methods:**

A mixed-methods approach was used, combining quantitative assessment using the Technology Acceptance Model 3 (TAM 3) and the System Usability Scale (SUS), with qualitative insights from a semistructured interview. Fifteen nursing staff members from a pediatric rheumatology clinic in Germany participated.

**Results:**

The PPR 2.0 Calculator was rated highly usable, with strong scores for Perceived Ease of Use (4.00) and Computer Self-Efficacy (4.09). Participants required minimal technical support, indicating an intuitive interface. However, Perceived Usefulness (2.82) and Job Relevance (2.53) scores were lower, suggesting limited value in daily workflows. The SUS score (65.50) was slightly below the benchmark of 68, indicating good usability with moderate room for improvement.

**Conclusion:**

Digitizing the analog PPR 2.0 catalog resulted in good usability, but significant challenges regarding practical relevance and workflow integration remained. Directly adopting the catalog content negatively affected perceived usefulness and job relevance, revealing limitations in the classification framework itself. Refinement of the PPR 2.0 framework is needed to reflect real-world clinical nursing tasks. Seamless integration with existing infrastructures and structured documentation is also critical. Future improvements should go beyond simple digitization and explore automated classification features.

## Background and Significance

To address the nursing staffing crisis, the German Federal Ministry of Health introduced the Pflegepersonalbemessungsverordung (PPBV), implementing the Pflegepersonalregelung 2.0 (PPR 2.0) framework in January 2024. 1 The primary objective of PPR 2.0 is to ensure adequate staffing levels for patient care by establishing standardized guidelines for determining nursing personnel requirements. PPR 2.0 is mandatory for staffing measurements in general wards for adults, general pediatric wards, and pediatric intensive care units. It provides regulations on the minimum number of nursing staff required per shift in hospital wards. To determine the number of nurses required for a ward, nursing staff must classify each patient daily into two care level categories: General Care (A1–A4) and Specialized Care (S1–S4). Each category is assigned a specific time value quantifying the required nursing effort. The sum of all patient time values determines the overall nursing staff needs. To classify patients into care levels, the PPR 2.0 framework provides a comprehensive classification catalog to guide nursing staff. However, this daily classification process is time-consuming. A study commissioned by the German Federal Ministry of Health found that in adult wards, classification takes an estimated 1 to 2 minutes per patient, while in pediatric wards, the process takes approximately 5 minutes per patient. 2 In a 30-bed ward, this amounts to nearly 3 hours of documentation per day. The administrative burden reduces the time available for direct patient care. Implementing digital technology, such as the PPR 2.0 Calculator, could streamline the classification process, reduce documentation time, and improve accuracy. However, the tool must be user-friendly and widely accepted in clinical practice to ensure that it effectively supports nursing staff.

The Technology Acceptance Model (TAM) is widely used to examine technology adoption in nursing. 3 At its core, TAM emphasizes Perceived Usefulness and Perceived Ease of Use as the primary determinants of user acceptance. TAM 3 4 is one of the most comprehensive adaptations of the model. Reviews from 2018 5 and 2021 6 showed that TAM and its extensions are reliable and have been widely applied in the nursing field, particularly in evaluating the adoption of telemedicine applications, electronic health records and electronic medical records, mobile or web applications. Additionally, TAM's application extends to assessing artificial intelligence (AI), 7 8 augmented reality (AR), 9 and robotics applications 10 11 in clinical nursing. Alongside TAM, the System Usability Scale (SUS) 12 is a widely used metric that provides a reliable and standardized measure of system usability with a typically high Cronbach's α coefficient (often exceeding 0.90). 13 A scoping review from 2019 14 identified SUS as the most frequently used questionnaire for evaluating usability of digital health applications. Despite extensive research on digital technology usability and acceptance in nursing, no studies have specifically evaluated digital tools for PPR 2.0 implementation.

## Objectives

Despite extensive research on digital technology usability and acceptance in nursing, no studies have specifically evaluated digital tools designed for PPR 2.0 implementation. Therefore, the aim of this pilot study is to investigate whether a direct digitization of the analog PPR 2.0 classification catalog into a digital user interface provides practical benefits. By assessing the acceptance and usability of the PPR 2.0 Calculator, this study offers quantitative and qualitative insights from the perspective of nursing staff. This leads to the following research question: How do nursing staff perceive the acceptance and usability of a web-based application that digitizes the analog PPR 2.0 classification catalog for use in clinical nursing practice?

## Methods

### Development of PPR 2.0 Calculator

**Fig. 1:**
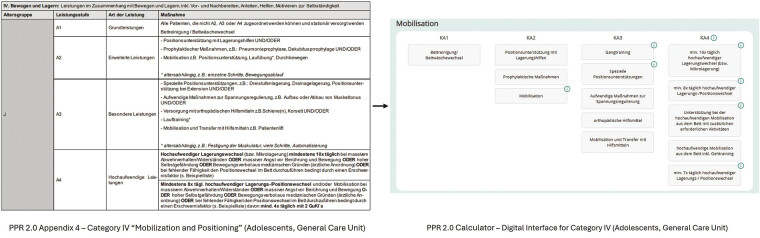
Digital implementation of the legally defined PPR 2.0 classification catalog. The figure shows category IV “Mobilization and Positioning” for adolescent patients in general care. The original regulatory text (left) was directly adopted without modification and represented as selectable elements in the user interface of the PPR 2.0 Calculator (right), preserving the terminology and structure defined by law. PPR 2.0, Pflegepersonalregelung 2.0.

The PPR 2.0 Calculator digitizes the entire PPR 2.0 classification catalog, translating it directly into a digital user interface ( Fig. 1 ). The structure of the containers within the application precisely mirrors the categories outlined in the original PPR 2.0 framework. Similarly, all content within these containers and the buttons used for selection are fully based on the original content provided by the PPR 2.0 classification system. Nursing staff can determine a patient's care level with just a few clicks by selecting the performed care measures, with the system automatically calculating the required nursing minutes and providing the corresponding care level in real-time. In contrast, the analog catalog requires nursing staff to manually search for performed care measures in the catalog and then read out the classification.

**Fig. 2:**
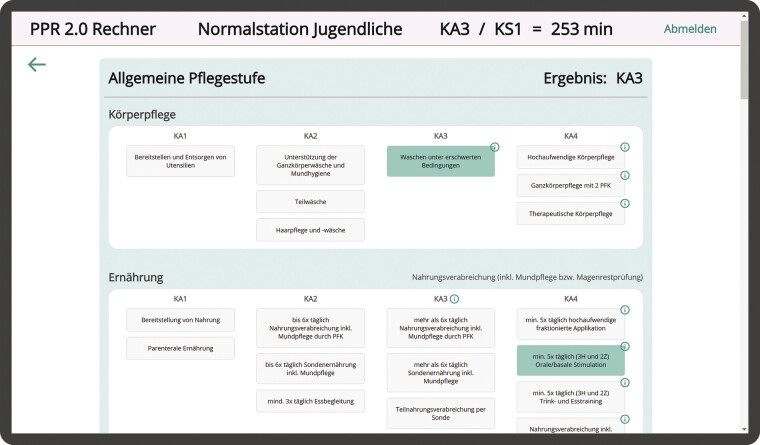
PPR 2.0 Calculator view—general pediatric wards. As selections are made, the system automatically updates and displays the patient's care level (KA3/KS1) and the calculated minutes value in the header in real-time. PPR 2.0, Pflegepersonalregelung 2.0.

Implemented as a progressive web app using JavaScript, the PPR 2.0 Calculator offers platform independence and does not require installation, accessible directly through browsers on any device. The application requires an internet connection and is accessible exclusively online. Additionally, the application calculates patient classifications based solely on selected care activities, without processing or storing any personal health data. Results are not linked to identifiers and are manually transferred into the hospital information system (HIS) by nursing staff. The application is, therefore, General Data Protection Regulation (GDPR)-compliant and does not require a data protection agreement. The calculator is accessed at pflegeberechnung.de, where users can log in, select the nursing care sector, and then the interface for calculating patient care levels is displayed ( Fig. 2 ).

### Study Design

This pilot study employed quantitative and qualitative approaches to evaluate the acceptance and usability of the PPR 2.0 Calculator. The quantitative component utilized questionnaires based on the TAM 3 4 and SUS. 12 The qualitative component consisted of a semistructured interview to gather in-depth information.

### Study Setting and Participants

The study was conducted in a pediatric rheumatology acute care clinic in Germany, which treats more than 1,500 inpatients annually. The study setting provided a realistic environment for assessing the calculator's performance in routine clinical workflows, including both day and night shifts. A total of 15 nurses from six different departments participated in the quantitative survey, while 1 nurse participated in the qualitative interview. After a brief introduction by nursing management, participants used the PPR 2.0 Calculator during the study period from September 16 to September 30, 2024.

### Data Collection

Data collection was conducted after the study period. The quantitative data were collected through standardized questionnaires, which were administered online using Microsoft Forms ( *https://forms.office.com/* ). For all questionnaires, German versions were used (local language). A link to the questionnaires was sent to the participating nursing staff via email. On average, completing the questionnaires took 6 minutes and 20 seconds. Demographic data (gender, age, and nursing experience) were collected at the beginning of the questionnaire. Digital literacy was also included as an item and assessed through a self-rating by participants on a 5-point Likert scale ranging from “no experience” to “very good.” The technology acceptance evaluation applied a modified TAM 3 questionnaire, including the constructs Perceived Usefulness (PU), Perceived Ease of Use (PEOU), Computer Self-Efficacy (CSE), Perceptions of External Control (PEC), Computer Anxiety (CANX), Job Relevance (REL), Output Quality (OUT), Result Demonstrability (RES), and Behavioral Intention (BI). The items for the original TAM model factors 3 were adapted for PU and PEAU evaluation. The items for all other subscales were newly formulated. The items can be seen in Supplementary Material S1 (available in the online version only). The SUS was employed as a standardized measure to assess the usability of the PPR 2.0 Calculator from the perspective of nursing staff. The SUS comprises 10 subjective assessment items, including 5 positive and 5 negative items, using a 5-point Likert scale. The qualitative component involved a semistructured interview with one participant, focusing on their experiences using the PPR 2.0 Calculator. The interview was conducted via telephone call using predefined questions to ensure consistency and comparability. Notes were taken during the conversation. For the interview questionnaire, a German version was used (local language). The interview was structured around the core constructs of the modified TAM 3 questionnaire, with specific questions addressing each construct ( Table 1 ).

**Table 1:** Semistructured interview questions

ID	Question
Q1.	In what ways is the PPR 2.0 Calculator useful for patient classification?
Q2.	What do you think about the usability of the calculator, and would you suggest any changes?
Q3.	Do you feel confident using the PPR 2.0 Calculator independently?
Q4.	How much time do you have to use the calculator, and where do you typically use it?
Q5.	How did you feel while using the calculator—secure, stressed, or intimidated?
Q6.	How relevant is the PPR 2.0 Calculator to your daily work?
Q7.	Do you trust the results of the calculator, and what additional results would you like to see at the end of the calculation?
Q8.	How understandable and transparent are the results provided by the calculator?
Q9.	How frequently do you plan to use the PPR 2.0 Calculator in the future?

Abbreviation: PPR 2.0, Pflegepersonalregelung 2.0.

### Data Analysis

Quantitative data collected through TAM 3 and SUS questionnaires were analyzed using descriptive statistics in Microsoft Excel (Microsoft Excel für Microsoft 365 MSO Version 2501). The TAM 3 questionnaire was analyzed by calculating the mean (M) and standard deviation (SD) of each item and construct. Reversed scoring was applied to negatively worded items to ensure consistency, and an overall TAM 3 score was computed by averaging the scores of all constructs. Cronbach's α was used to assess the consistency of the modified TAM 3 questionnaire. Pearson's correlations were used to determine the correlation between the investigated constructs. For the SUS analysis, both the overall SUS score and individual item means were calculated. 12

## Results

### Participant Characteristics

The study included 15 female nurses with diverse ages, experience levels, and digital literacy. Most were under 25, while a smaller proportion were older. Experience ranged from less than 2 years to over a decade. Digital literacy was generally rated as good or very good. This diversity allowed for a comprehensive evaluation of the PPR 2.0 Calculator. Participant characteristics are detailed in Table 2 .

**Table 2:** Characteristics of the participants ( *n* = 15)

Characteristics	Value	Frequency	Percentage
Gender	Women	15	100
Age (y)	≤25	7	46.7
	36–45	4	26.7
	46–55	2	13.3
	56–65	2	13.3
Nursing experience (y)	1–2	4	26.7
	3–5	3	20
	6–10	2	13.3
	>10	6	40
Digital literacy	Very good	6	40
	Good	4	26.7
	Average	5	33.3

The correlation analysis presents relationships between the constructs, as shown in Table 3 . The strongest correlations were observed between PU and BI ( *r* = 0.84), RES and OUT ( *r* = 0.84), OUT and BI ( *r* = 0.82), and RES and BI ( *r* = 0.75). Moderate correlations were found between PU and OUT ( *r* = 0.71), PU and RES ( *r* = 0.68), CSE and CANX ( *r* = 0.67), REL and OUT ( *r* = 0.67), REL and PU ( *r* = 0.57), RES and PEOU ( *r* = 0.54), and REL and BI ( *r* = 0.54).

**Table 3:** Correlations between Technology Acceptance Model 3 subscales

Construct	PU	PEUO	CSE	PEC	CANX	REL	OUT	RES	BI
PU	–	–	–	–	–	–	–	–	–
PEUO	0.25	–	–	–	–	–	–	–	–
CSE	0.30	0.36	–	–	–	–	–	–	–
PEC	0.36	0.30	0.41	–	–	–	–	–	–
CANX	0.46	0.13	0.67	0.53	–	–	–	–	–
REL	0.57	0.03	−0.17	0.23	0.02	–	–	–	–
OUT	0.71	0.49	0.09	0.33	0.11	0.67	–	–	–
RES	0.68	0.54	0.19	0.41	0.16	0.60	0.84	–	–
BI	0.84	0.24	0.17	0.29	0.45	0.54	0.82	0.75	–

Abbreviations: BI, Behavioral Intention; CANX, Computer Anxiety; CSE, Computer Self-Efficacy; OUT, Output Quality; PEC, Perceptions of External Control; PEOU, Perceived Ease of Use; PU, Perceived Usefulness; REL, Job Relevance; RES, Result Demonstrability.

### Technology Acceptance

Table 4 presents the descriptive statistics for the TAM 3 subscales. The highest mean scores were observed for CSE (M = 4.09, SD = 1.85), PEOU (M = 4.00, SD = 0.60), and PEC (M = 3.98, SD = 0.75). In contrast, the lowest mean scores were reported for REL (M = 2.53, SD = 0.86), PU (M = 2.82, SD = 0.84), and OUT (M = 2.89, SD = 1.09). Moderate scores were found for CANX (M = 3.57, SD = 1.36), RES (M = 3.40, SD = 0.97), and BI (M = 3.11, SD = 1.01). The internal consistency of the TAM 3 constructs, measured using Cronbach's α, is reported for each subscale. The highest reliability was found for BI (α = 0.91). PEC (α = 0.86), OUT (α = 0.86), PU (α = 0.84), and REL (α = 0.83) also showed high reliability. PEOU (α = 0.70) and CSE (α = 0.74) demonstrated acceptable reliability. CANX (α = 0.63) and RES (α = 0.62) had lower internal consistency.

**Table 4:** Acceptance of the PPR 2.0 Calculator based on Technology Acceptance Model 3 ( *n* = 15)

Constructs a	Mean (SD)	Cronbach's α
PU	2.82 (0.84)	0.84
PEOU	4.00 (0.60)	0.70
CSE	4.09 (1.85)	0.74
PEC	3.98 (0.75)	0.86
CANX	3.57 (1.18)	0.63
REL	2.53 (0.86)	0.83
OUT	2.89 (0.96)	0.86
RES	3.40 (0.81)	0.62
BI	3.11 (1.01)	0.91

Abbreviations: BI, Behavioral Intention; CANX, Computer Anxiety; CSE, Computer Self-Efficacy; OUT, Output Quality; PEC, Perceptions of External Control; PEOU, Perceived Ease of Use; PU, Perceived Usefulness; REL, Job Relevance; RES, Result Demonstrability; SD, standard deviation.

a Responses were scored on a 5-point Likert scale ranging from 1 (strongly disagree) to 5 (strongly agree).

### Usability

The total SUS score of 65.50 (SD = 0.44) falls slightly below the general industry benchmark of 68, 15 which can be used when evaluating digital health applications. 13 Individual item scores are reported in Table 5 . The highest ratings were observed for technical support (M = 3.27, SD = 0.59), ease of use (M = 3.07, SD = 0.80), and learnability (M = 3.00, SD = 0.65), while the lowest scores were found for frequency of use (M = 2.07, SD = 1.10) and integration of system functions (M = 2.00, SD = 1.00).

**Table 5:** System Usability Scale scores for PPR 2.0 Calculator evaluation ( *n* = 15)

SUS item a	Mean (SD)
1. Frequency of use: Likelihood of frequent system use	2.07 (1.10)
2. Complexity: System's perceived complexity	2.40 (1.25)
3. Ease of use: Ease with which users can operate the system	3.07 (0.80)
4. Technical support: Need for technical support	3.27 (0.59)
5. Integration: Integration of system functions	2.00 (1.00)
6. Consistency: System's consistency in performance	2.20 (0.86)
7. Learnability: Users' ability to quickly learn the system	3.00 (0.65)
8. Cumbersomeness: The cumbersome nature of the system	2.73 (1.16)
9. Confidence: Confidence level of users while using the system	2.60 (0.91)
10. Prior learning: Amount of learning required before use	2.87 (1.13)
Total System Usability Scale score	65.50 (0.44)

Abbreviations: SD, standard deviation; SUS, System Usability Scale.

a Responses were scored on a 5-point Likert scale ranging from 0 (strongly disagree) to 4 (strongly agree).

### Interview Results

Prior to the interview, the participating nurse reported that staff from all six involved units had discussed the interview topics internally. As such, the views expressed in the interview may reflect a shared understanding among the nursing teams. The participant found the system useful for patient classification but noted challenges with system integration and patient care level classification. One of the primary issues noted was the lack of integration with the HIS. Since care measures needed for classification are documented in the HIS, the inability to have both systems open simultaneously posed a significant challenge. Constantly switching between systems disrupted workflow efficiency and was perceived as a limitation. Additionally, regarding REL, the participant noted that the calculator had limited applicability in daily work. Some users experienced uncertainty when selecting specific fields for patient care level classification, as the available care measures in the calculator did not always align with the actual tasks performed. This issue was particularly relevant in the pediatric rheumatology clinic, where therapeutic interventions are the primary responsibility of nurses. However, since PPR 2.0 does not account for therapeutic interventions, the classification process sometimes felt less applicable to the clinic's daily operations. For instance, gait training, which is performed as part of patient care, was not recognized as a nursing activity, leading to ambiguity in the classification process. To enhance acceptance and usability, it was suggested that clearer differentiation between nursing and therapeutic tasks could improve the system's applicability in clinical settings where therapeutic care is prevalent. Additionally, it was recommended that staff receive better training on how to document deviations from standard care practices. Despite these concerns, the PPR 2.0 Calculator was generally described as clear, straightforward, and easy to navigate. The real-time feedback feature, which updates the patient's care level instantly as selections are made, was particularly appreciated. The participant expressed high confidence in using the system independently, emphasizing that its design was self-explanatory and required minimal guidance or prior training. Regarding PEC, the participant stated that the PPR 2.0 Calculator was primarily used during night shifts, where free access to computers was available in the station's office. The participant also emphasized that there was sufficient time to use the system without significant disruptions to workflow. When asked about CANX, the participant reported no significant concerns, stating that initial uncertainty quickly subsided as they became familiar with the interface. In terms of OUT, the participant expressed trust in the accuracy of the calculator's results, stating that the system reliably generated classifications based on selected care measures. Similarly, RES was rated positively, with the participant describing the calculator's results as transparent and easy to understand. Regarding BI, the participant stated that they intended to use the calculator daily but questioned its usefulness during night shifts. Since night shift staff often relied on retrospective documentation to classify care measures performed by previous shifts, some important details were lost in the process. Specific care activities were often documented only in free-text notes rather than in structured care plans, making it difficult for night shift staff to accurately assess the patient's care level. The participant suggested that the system would be more effective if each shift performed its own classification. In addition to these observations, the participant also reported that the PPR 2.0 Calculator continues to be actively used on all six participating wards after the study ended. This ongoing use suggests that, despite certain usability and integration challenges, the tool is considered more practical and preferable compared to the original paper-based classification catalog.

## Discussion

### Usablilty and Ease of Use

The high ratings for PEOU and CSE suggest that the system was intuitive and required minimal training. This is further supported by the low need for technical support. The majority of participants rated their digital literacy as “good” or “very good,” indicating that low digital literacy was unlikely to have biased the usability evaluations. Furthermore, the SUS score confirmed that the system was perceived as usable, though slightly below the benchmark, indicating room for improvement in user satisfaction and performance. A closer examination of the individual SUS items provides further insight into specific areas for improvement. The lowest-rated items were frequency of use and integration of system functions. These results suggest that the tool was not perceived as fully embedded into the daily workflow, which may have affected routine use. This issue is explored further below. This finding aligned with the qualitative interview, where the participant described the system as clear, self-explanatory, and easy to use. Minimal support was required, and confidence in using the tool was high, even without formal training. Furthermore, the high PEC score indicates that users felt they had sufficient resources and support to effectively use the system, suggesting that technical barriers were not a major concern. Although the application is accessible exclusively online, this did not negatively impact usability or acceptance in the study setting, as a reliable internet connection was available throughout the entire facility. The good usability results indicate that structuring the containers of the web application according to the categories of the PPR 2.0 and displaying contents as buttons within these containers effectively supports ease of use.

### System Relevance and Adoption

Despite its usability, moderate PU and REL scores indicate doubts about its value in daily tasks. The low rating for frequency of use in the SUS assessment further supports this finding, indicating that users did not tend to use the calculator frequently. This is consistent with the strong correlation between PU and BI, which suggests that users were only likely to continue using the system if they perceived it as beneficial for their work. This is further reflected in the strong correlation between PU and OUT and between PU and RES, indicating that users who found the system's outputs reliable and well-presented were also more likely to find it useful. Conversely, the weak correlation between PEOU and BI suggests that ease of use alone does not directly influence adoption. Instead, other factors, such as usefulness and output quality, appear to play a more decisive role in whether users integrate the system into their daily workflow. Interestingly, despite the moderate scores for perceived usefulness and frequency of use, the PPR 2.0 Calculator continues to be actively used across all six participating wards. This information emerged from the qualitative interview, in which the participant confirmed that the tool remained in daily use beyond the study period. This suggests that, in real-world conditions, the digital application is still perceived as more practical and manageable than the analog paper-based catalog. Its continued use highlights a pragmatic acceptance by nursing staff and indicates that, despite current limitations, the tool provides meaningful support for completing PPR 2.0 classifications.

### Framework-Related Limitations

The qualitative findings helped to contextualize several quantitative results, particularly the lower scores for PU and REL. The interviewee reported difficulties when classifying certain activities based on the available options in the digital interface. This applied especially to therapeutic interventions such as gait training, which are central to nursing practice in the pediatric rheumatology setting. These activities could not be mapped to any category in the system, resulting in uncertainty and reduced perceived relevance. These classification issues appear to stem from the PPR 2.0 classification catalog itself, since the contents of the buttons were directly adopted from the original analog framework. The participant also noted that similar limitations had been encountered when using the paper-based version, suggesting that these problems are inherent to the framework rather than the result of digitization. The current PPR 2.0 framework does not sufficiently accommodate therapeutic interventions. These findings align with broader criticisms of the PPR 2.0 framework reported in the literature. 2 A key concern is that the current classification model focuses primarily on physical nursing activities, while other essential nursing tasks, such as patient education and psychosocial support, are not adequately represented. Additionally, terminological ambiguities within PPR 2.0—such as the interpretation of terms like “frequent” or “complex” interventions—have been identified in the interview as a major source of inconsistent patient classifications. This observation is also consistent with weaknesses reported in the literature regarding the clarity and operationalization of PPR 2.0 terminology. 2 As the PPR 2.0 Calculator directly digitizes the analog catalog without modification, these perceived limitations can be attributed to the original classification logic rather than the digital interface itself. The misalignment between daily nursing tasks and the PPR 2.0 classification system likely contributed to nurses perceiving the system as less useful and relevant to their daily work. To address these limitations, the PPR 2.0 framework may require targeted refinement to improve its applicability in different clinical environments. Specifically, the classification catalog should be expanded to include nursing activities relevant to specialized settings, such as therapeutic interventions in pediatric care. In addition, ambiguous terminology, such as “frequent” or “complex” interventions, should be clearly defined to reduce inconsistencies in classification. Finally, aligning the classification logic with structured nursing documentation systems could support more consistent and accurate use in daily practice.

### Documentation and Workflow Challenges

The qualitative interview also revealed challenges related to retrospective classification, especially during night shifts. Since classification was performed by staff who had not directly provided patient care, the risk of missing or inaccurate documentation increased. This issue was further compounded by the lack of a standardized documentation system in the study hospital. As a result, care activities were recorded in free-text fields rather than structured formats, making it difficult to retrieve relevant information for PPR 2.0 classification. Implementing a standardized system for documenting care measures, such as Leistungserfassung in der Pflege, 16 would ensure that all relevant information is systematically recorded and easily accessible for classification. Furthermore, aligning PPR 2.0 terminology with daily nursing documentation would reduce ambiguity and ensure documented care activities are easily mapped to classification fields.

### System Integration Challenges

One of the most significant barriers to routine use was the lack of integration with the HIS. Because patient care measures were documented in the HIS, users had to perform classification separately in the PPR 2.0 Calculator. This required constant switching between systems, especially problematic with single-screen setups. This created inefficiencies and increased cognitive workload, which may have contributed to lower PU and REL ratings. The low SUS scores for system integration and frequency of use support this interpretation. According to the interview participant, the inability to work in parallel with the HIS caused delays and interruptions. A possible solution would be to implement the PPR 2.0 Calculator directly as a functional module within the HIS. This would allow users to classify patient care levels without switching between systems, reduce workflow interruptions, and ensure that relevant information is accessible in one central environment. Such integration could significantly improve both usability and frequency of use by embedding the tool into routine clinical processes. A long-term improvement could involve moving beyond simple digitization of the classification catalog and instead developing an automated classification tool. By integrating natural language processing (NLP), 17 the tool could autoclassify patient care levels from nursing documentation in the HIS and assign PPR 2.0 patient care level classifications. This would eliminate the need for retrospective manual classification, ensuring that no critical information is lost between shifts.

### Limitations

This study has several limitations. First, the sample size was small ( *n* = 15), as the study was conducted as a pilot within a single highly specialized pediatric rheumatology clinic. This naturally restricted the number of eligible participants. While the small sample size limits the statistical generalizability of the findings, it aligns with the exploratory nature of a pilot study and supports the aim of generating initial insights into the usability and acceptance of the PPR 2.0 Calculator in a real-world clinical setting. Second, all participants were female. This reflects the actual staff composition of the six participating nursing units, where no male nurses were employed at the time of data collection. In the German hospital system, nursing continues to be a predominantly female profession, particularly in inpatient care. Therefore, the lack of gender diversity in the sample is not the result of sampling bias and does not compromise the validity of the findings within the given study context. Third, the qualitative component was limited to a single interview, due to voluntary participation and time constraints. The resulting qualitative data should be interpreted as exploratory and illustrative rather than representative. However, the interview participant reported that nursing staff from all six participating units had coordinated their views in advance, suggesting that the shared insights may reflect a collective perspective rather than an individual opinion. Fourth, this pilot study focused exclusively on evaluating usability and acceptance. It did not examine effects on classification accuracy, time efficiency, or clinical outcomes. These aspects will be addressed in future studies. Fifth, the study applied TAM 3 as the sole theoretical framework to assess technology acceptance. While TAM 3 provides a robust basis for evaluating perceived usefulness and usability, it does not account for contextual factors such as organizational support, peer influence, or system-level policies. Future studies may benefit from integrating complementary models, such as the Unified Theory of Acceptance and Use of Technology (UTAUT), 18 to capture these additional dimensions. Sixth, the study was conducted in a pediatric rheumatology clinic, where therapeutic interventions play a central role. Therefore, the findings on job relevance and perceived usefulness may not fully apply to hospitals with a stronger focus on standard nursing tasks. Finally, the study examined only initial usability and acceptance, without assessing long-term adoption. To address these limitations, future research should expand the evaluation of the PPR 2.0 Calculator to different hospital departments, such as general medical wards and intensive care units, where PPR 2.0 classifications may be more relevant. Longitudinal studies with larger and more diverse participant groups are needed to better understand factors influencing sustained use.

## Conclusion

This study contributes to the growing body of research on digital tool adoption in nursing by evaluating the PPR 2.0 Calculator, a digital tool that directly digitizes the analog PPR 2.0 classification catalog into a structured user interface. By applying the TAM 3 and SUS frameworks alongside qualitative insights, the study shows that good usability alone does not guarantee successful adoption. Perceived usefulness and alignment with daily nursing practice remain critical for integration into routine workflows. Importantly, the findings indicate that many of the tool's perceived limitations stem from the underlying PPR 2.0 framework rather than the digital interface itself. Despite these constraints, the PPR 2.0 Calculator demonstrated promising potential for streamlining classification processes in clinical practice. Notably, the tool continues to be actively used across all six participating wards, as confirmed in the qualitative interview, which highlights its practical utility and perceived advantages over the traditional paper-based approach. Beyond administrative efficiency, the tool may also contribute to improved patient care by supporting more accurate and consistent patient care level classifications. Under the PPR 2.0 system, these classifications serve as the basis for calculating the required number of nursing staff per shift. Therefore, improving the accuracy and consistency of classifications through digital support may help ensure more appropriate staffing, reduce the risk of understaffing, and better align available resources with actual patient needs. In the context of PPR 2.0 regulations, where hospitals face financial and legal consequences if calculated staffing requirements are not met, a digital tool that promotes classification consistency may also enhance regulatory compliance.

The study provides practical guidance for system integration, user-centered design, and the refinement of classification frameworks to better reflect the realities of modern nursing. Future research should explore whether tools like the PPR 2.0 Calculator lead to more reliable patient classifications and improved care outcomes, and whether automated approaches could further increase efficiency and applicability in diverse clinical settings.

## Clinical Relevance Statement

The direct digital implementation of the analog PPR 2.0 classification catalog resulted in good usability but lacked practical relevance for nursing staff. Our findings suggest that classification frameworks like PPR 2.0 must be adapted to reflect the realities of specialized nursing environments, including therapeutic and activities, and that standardizing nursing documentation terminology could enhance interoperability and improve alignment with classification systems. Future improvements should go beyond simple digitization and focus on integrating tools into HISs and ensuring automated classification features.

## Multiple-Choice Questions

1. What did the study reveal about the relationship between usability and long-term adoption of the PPR 2.0 Calculator?
  - High usability automatically ensured long-term use.
  - Perceived usefulness was more important than usability for adoption.
  - Usability scores were irrelevant to behavioral intention.
  - Technical support was the primary factor in continued use.
  **Correct Answer** : The correct answer is option b. The study found that ease of use alone was not a sufficient driver of behavioral intention to use the system long-term. Strong correlations were found between perceived usefulness, job relevance, and behavioral intention, indicating that practical benefit in daily routines outweighed usability.
2. Which of the following improvements were suggested to enhance the clinical relevance and adoption of the PPR 2.0 Calculator?
  - Replace the nursing staff with administrative users
  - Translate the tool into five additional languages
  - Embed automated classification features into the tool
  - Convert the system into a mobile-only app
  **Correct Answer** : The correct answer is option c. The study suggests that future versions of the PPR 2.0 Calculator should move beyond manual digitization and incorporate automated classification. Technologies such as NLP could analyze structured and unstructured nursing documentation in real time and suggest appropriate care levels, minimizing manual input and errors.
