# Supplementary material for: Acceptance and Usability of a Web Application for Patient Care Level Classification in German Clinical Nursing Care: A Pilot Study
Source: Appl Clin Inform. 2025 Dec 11;16(5):1828–36. doi: 10.1055/a-2753-9439 (PMC12698287; doi:10.1055/a-2753-9439)
Supplement: Supplementary file 1 — Supplementary Material [file 10-1055-a-2753-9439_27700530.pdf]

Thieme

Supplementary Material S1

Perceived Usefulness (PU)

Item No.	Item
1	The PPR 2.0 Calculator helps me complete my work tasks more quickly.
2	The PPR 2.0 Calculator helps me make fewer mistakes in my work tasks.
3	Using the PPR 2.0 Calculator makes it easier to complete my daily work.
4	Overall, I find the PPR 2.0 Calculator useful for my daily work.

Perceived Ease of Use (PEOU)

Item No.	Item
1	I find the PPR 2.0 Calculator easy to use.
2	I think the PPR 2.0 Calculator is easy to learn.
3	Using the PPR 2.0 Calculator requires little effort.

Computer Self-Efficacy (CSE)

Item No.	Item
1	I believe I can use the PPR 2.0 Calculator independently once it has been explained to me.
2	I believe I can use the PPR 2.0 Calculator independently even without prior explanation.
3	I believe I can only use the PPR 2.0 Calculator with help from others.

Perceptions of External Control (PEC)

Item No.	Item
1	The technical infrastructure required to use the PPR 2.0 Calculator is available to me.
2	I have the necessary time to use the PPR 2.0 Calculator effectively.
3	I have the necessary space/workstation to use the PPR 2.0 Calculator effectively.

Computer Anxiety (CANX)

Item No.	Item
1	I am afraid of making mistakes when using the PPR 2.0 Calculator.
2	I feel stressed when I have to use the PPR 2.0 Calculator.

Job Relevance (REL)

Item No.	Item
1	The PPR 2.0 Calculator is important for completing my daily tasks.
2	The PPR 2.0 Calculator is a necessary tool to fulfill my daily tasks.
3	The functions of the PPR 2.0 Calculator are directly applicable to my daily tasks.

Output Quality (OUT)

Item No.	Item

1	I trust the output provided by the PPR 2.0 Calculator.
2	I am satisfied with the accuracy of the information provided by the PPR 2.0 Calculator.
3	The PPR 2.0 Calculator delivers consistent results with the same input.

Result Demonstrability (RES)

Item No.	Item
1	The results of the PPR 2.0 Calculator are traceable and transparent.
2	It is easy to demonstrate the benefits of the PPR 2.0 Calculator.

Behavioral Intention (BI)

Item No.	Item
1	I intend to use the PPR 2.0 Calculator regularly.
2	If possible, I will continue to use the PPR 2.0 Calculator in the future.
3	I would recommend the PPR 2.0 Calculator to my colleagues.
